# Identification of aminosulfonylarylisoxazole as microRNA-31 regulators

**DOI:** 10.1371/journal.pone.0182331

**Published:** 2017-08-04

**Authors:** Kyungtaek Im, Jiho Song, Young Taek Han, Seul Lee, Soowon Kang, Kwang Woo Hwang, Hyeyoung Min, Kyung Hoon Min

**Affiliations:** 1 College of Pharmacy, Chung-Ang University, Seoul, Korea; 2 College of Pharmacy, Dankook University, Chungnam, Korea; University of Toronto, CANADA

## Abstract

The discovery of small-molecule regulators of microRNAs remains challenging, but a few have been reported. Herein, we describe small-molecule inhibitors of miR-31, a tumor-associated microRNA (miRNA), identified by high-throughput screening using a cell-based reporter assay. Aminosulfonylarylisoxazole compounds exhibited higher specificity for miR-31 than for six other miRNAs, i.e., miR-15a, miR-16, miR-21, miR-92a-1, miR-146a, and miR-155, and increased the expression of miR-31 target genes. The down-regulation of mature miR-31 was observed, while its precursor form increased following treatment with the compounds. Thus, the compounds may target the processing of pre-miR-31 into mature miR-31 and thereby inhibit the production of mature miR-31.

## Introduction

MicroRNAs (miRNAs) are small noncoding RNAs that are 18–25 nucleotides in length and have been reported to function in post-transcriptional regulation of the expression of target mRNAs [1]. miRNAs are critical regulators of numerous cellular processes, and deregulated expression of some miRNAs has been observed in distinct types of cancers, implying their critical roles in tumorigenesis [2]. miR-31 is known to play roles in diverse biological processes, such as cell proliferation, apoptosis, migration, and cytokine production [3–5]. Aberrant expression of miR-31 has been reported in several diseases. In particular, miR-31 overexpression has been observed in esophageal squamous cell carcinoma [6] and colorectal [7, 8], oral [9], and lung cancers [10], suggesting an oncogenic role of miR-31. In contrast, miR-31 has also been reported to exert tumor suppressive effects in glioblastoma, lung adenocarcinoma, bladder cancer, and liver cancer [11–14]. In addition, miR-31 has been implicated in inflammatory and autoimmune diseases, including psoriasis, inflammatory bowel disease, and lupus [15–17]. Therefore, modulation of miR-31 expression can serve as a potential therapeutic strategy for various diseases associated with aberrant miR-31 expression.

Small molecule regulators have drawn considerable research attention because of their promising use in the regulation of miRNA expression [18]. Several studies have employed chemical screening for the discovery of small molecules that can regulate miRNA expression [19–21]. However, only a few studies have successfully identified regulators that can selectively control specific miRNAs [20, 21]. Instead, most studies have identified universal inhibitors or activators of miRNA expression [22–27]. Selectivity of a compound for a specific miRNA might be essential for its use in drug development or as a research tool.

To identify specific regulators of miR-31, we established a small-molecule screening system based on a secreted alkaline phosphatase (SEAP) reporter construct [28] instead of the luciferase reporter system [20, 21]. To screen for compounds that exhibit miR-31-modulating activity, we designed a SEAP reporter construct containing a complementary sequence to miR-31 in the 3′UTR of the *SEAP* gene, such that miR-31 expression levels are inversely related to *SEAP* expression. Accordingly, the presence of mature miR-31 would result in reduced *SEAP* expression, whereas inhibition of miR-31 by small molecules would lead to increased *SEAP* activity.

Herein, we report the identification of small-molecule regulators of miR-31 using a simple screening method and investigate their corresponding modes of action.

## Materials and methods

### Cell culture

Human embryonic kidney cells (HEK-293T; American Type Culture Collection, Manassas, VA, USA) were cultured in Dulbecco’s modified Eagle medium (DMEM) and DMEM without phenol red (Welgene, Seoul, Korea) for the secreted alkaline phosphatase (SEAP) reporter assay. The media contained 10% fetal bovine serum (Welgene), 100 units/mL penicillin, and 100 μg/mL streptomycin (Invitrogen, Carlsbad, CA, USA). The A549 (human lung cancer cell line; Korean Cell Line Bank, Seoul, Korea) and MCF-7 (human breast cancer cell line; Korean Cell Line Bank) cells were cultured in RPMI1640 medium (Welgene) containing 10% fetal bovine serum, 100 units/mL penicillin, and 100 μg/mL streptomycin. Cells were cultured at 37°C in a 5% CO_2_ atmosphere.

### Construction of plasmids and miR-31 mimics

To construct the miRNA (miR-21-5p, miR-31-5p, miR-92a-1- 3p, miR-155, and miR-223-3p) expression plasmid, a DNA fragment containing the precursor miRNA sequence and an additional 100 bp of flanking sequence was amplified and subsequently cloned into the MDH-PGK-GFP_2.0 vector. To produce the miRNA target fragment, sense and antisense oligonucleotides were synthesized (Bioneer, Daejeon, Korea) with the appropriate restriction enzyme sites and annealed. The resulting fragment was cloned into the pGL3UC luciferase reporter vector, after which the luciferase expression region was replaced with the secreted alkaline phosphatase expression gene from the pSEAP2-control vector (Clontech, Mountain View, CA, USA). Mature miR-31 mimics and scrambled miRNA mimics were purchased from Bioneer.

### Secreted alkaline phosphatase (SEAP) reporter assay

To analyze reporter activity, HEK-293T cells were transiently transfected with a mixture of the miRNA expression plasmid and target reporter plasmid containing the *SEAP* gene using branched polyethylenimine (Sigma-Aldrich, St. Louis, MO, USA) and then incubated for 18 h. Cells were then re-seeded at 5 × 10^4^ cells per well in a 96-well plate in complete DMEM without phenol red. After 6 h, compounds from an in-house chemical library were added at 5 μM, followed by incubation for 24 h. To detect SEAP activity, supernatants were collected, and 1 mg/mL 4-nitrophenyl phosphate disodium salt hexahydrate (Sigma-Aldrich) solution was used as a substrate for SEAP. Thereafter, SEAP activity was detected at a wavelength of 405 nm using a ThermoMax Plate Reader (Molecular Devices, Sunnyvale, CA, USA).

### Flow cytometric analysis

Transfected HEK-293T cells were harvested to evaluate plasmid transfection efficiency. Flow cytometric analysis was performed using FACSCalibur. Data were analyzed using BD CellQuest Pro^TM^ software (BD Biosciences, Franklin Lakes, NJ, USA). Plasmid transfection efficiency was evaluated based on green fluorescence protein (GFP) intensity.

### RNA isolation and cDNA synthesis

Expression levels of mature miRNA, precursor miRNA, and target mRNA were determined. Total RNA was extracted using QIAzol (Qiagen, Valencia, CA, USA) according to the manufacturer’s instructions. cDNAs were synthesized from total RNA. cDNA synthesis of mature miRNA and precursor miRNA was performed using miScript RT II Kit (Qiagen) following the manufacturer’s instructions. To determine target mRNA levels, cDNA was synthesized using oligo dT primers. Then, 50 μM oligo dT primers was annealed to 1 μg of total RNA in a total volume of 15 μL. The mixture was incubated at 72°C for 5 min and chilled on ice. Reverse transcription was performed after the addition of 10 μL of RT-mix containing 5× M-MLV Reverse Transcriptase Buffer (Promega, Madison, WI, USA), 10 mM dNTPs (Promega), 40 U/μL RNase inhibitor (Promega), and 200 U/μL M-MLV reverse transcriptase (Promega). The total reaction mixture (25 μL) was incubated at 42°C for 60 min, heated to 70°C for 10 min, and then stored at −20°C.

### Quantitative real-time PCR (qRT-PCR)

The miScript Primer Assay, miScript Precursor Assay, and miScript SYBR Green PCR Kits (Qiagen) were used to quantify mature miRNAs (miR-15a, 16, 21, 31, 92a-1, 146a, and 155) and precursor miRNAs (miR-31 and 16) following the manufacturer’s instructions. *RNU6* was used as a normalization control. Target mRNA expression levels were estimated using the miScript SYBR Green PCR Kit (Qiagen) following the manufacturer’s instructions. Detailed qRT-PCR procedures were performed as previously described [29]. Gene expression levels were normalized against those of *GAPDH*. The following primer sequences were used to amplify target mRNAs: *PPP2R2A* (sense 5′-TCGGATGTAAAATTCAGCCA-3′, antisense 5′-GATGCACCTGGTATGTTTCC-3′), *STK40* (sense 5′-CAGCACTACGTCATCAAGGAG-3′, antisense 5′-CGATGTGTCCTCTTGTTGAGC-3′), *E2F2* (sense 5′-CGTCCCTGAGTTCCCAACC-3′, antisense 5′-GCGAAGTGTCATACCGAGTCTT-3′), and *GAPDH* (sense 5′-AATGGTGAAGGTCGGTGTGAAC-3′, antisense 5′-GAAGATGGTGATGGGCTTCC-3′). All qRT-PCRs were performed in triplicate using the CFX Connect™ Real-Time PCR Detection System (Bio-Rad, Hercules, CA, USA).

### Western blotting

Proteins were extracted in cell lysis buffer (Thermo Fisher, Bothell, WA, USA). Total protein concentrations were calculated using the Pierce BCA Protein Assay Kit (Thermo Fisher). Then, 20 μg of cell lysates from A549 and MCF-7 cells were collected, separated by 10% SDS-PAGE, and transferred onto polyvinylidene fluoride membranes (Bio-Rad). PVDF membranes were blocked with TBS Tween-20 buffer containing 5% skim milk and incubated with the primary antibody at 4°C overnight with gentle shaking. After washing, horseradish peroxidase (HRP)-conjugated secondary antibodies (Cell Signaling, Beverly, MA, USA) were added, and the reaction was incubated at room temperature for 2 h. ECL solution (GenDepot, Barker, TX, USA) was added to the membrane. Detection was performed using the ChemiDoc™ XRS+ System (Bio-Rad).

### Statistical analysis

All experiments were repeated at least thrice. Data were expressed as mean ± standard deviation. Student’s *t*-tests were performed to determine significant differences among groups.

## Results

### Screening of compounds with miR-31-modulating activity

miR-21-5p, miR-31-5p, miR-92a-1- 3p, miR-155, and miR-223-3p are well-known oncogenic miRNAs. To identify specific regulators that stimulate or inhibit the expression of these oncogenic miRNAs, we conducted primary screening of hundreds of compounds from an in-house chemical library using HEK-293T cells at 5 μM. HEK-293T cells were co-transfected with two specifically designed plasmids, namely, an miRNA expression construct containing a GFP marker and a SEAP reporter construct containing the corresponding miRNA binding sites in the 3'UTR of the *SEAP* reporter gene. Compounds that stimulate miRNA expression are expected to inhibit the enzymatic activity of SEAP, whereas compounds that inhibit miRNA expression would increase SEAP activity. Flow cytometry based on GFP expression was used to measure transfection efficiency. To minimize the number of false negatives and false positives, we normalized SEAP activity with respect to cell viability and performed repeated experiments for primary hits.

**Fig 1:**
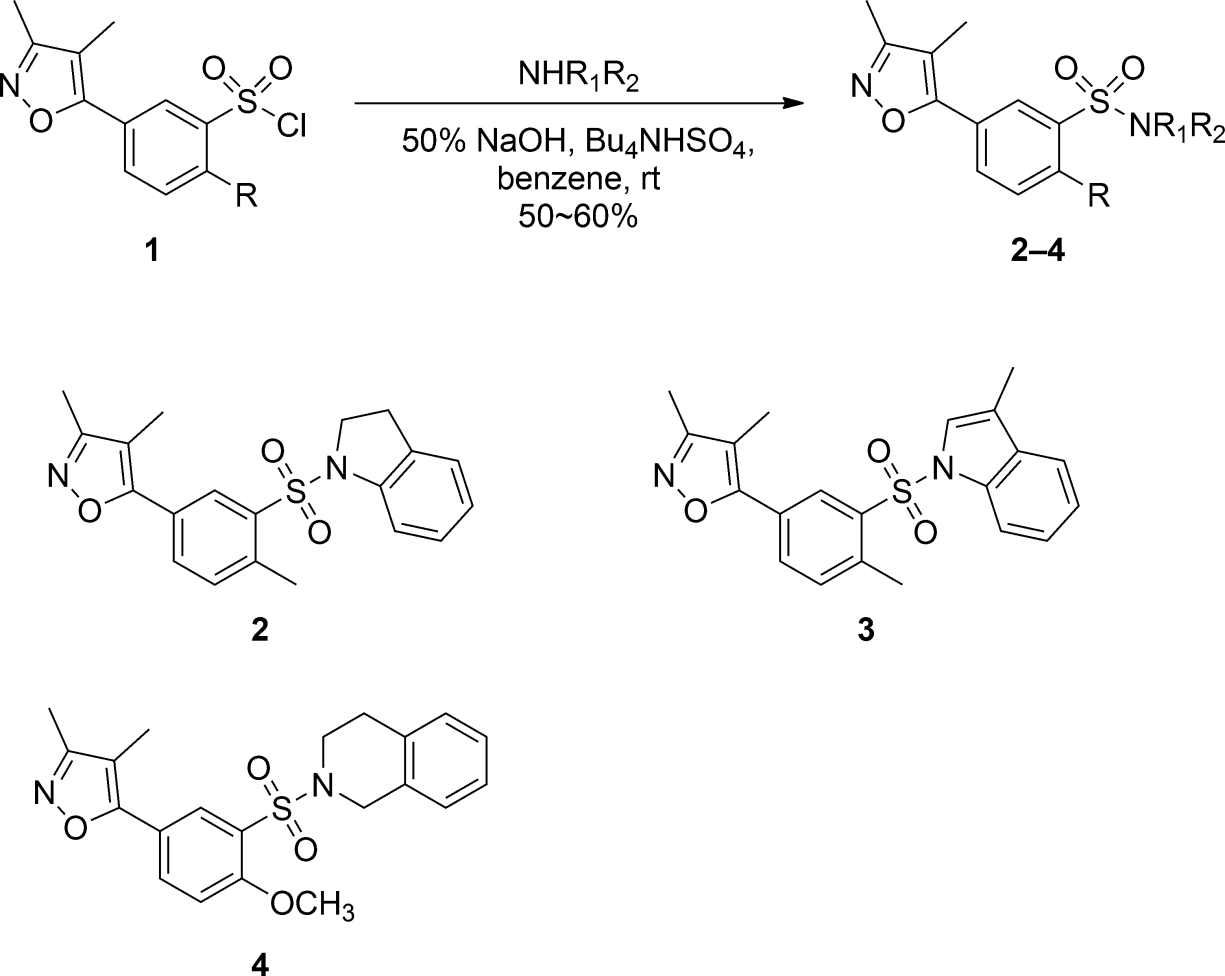
Synthesis of aminosulfonylarylisoxazole derivatives. Compounds **2–3** were prepared via the coupling reaction of sulfonyl chloride **1** and the corresponding amine.

**Fig 2:**
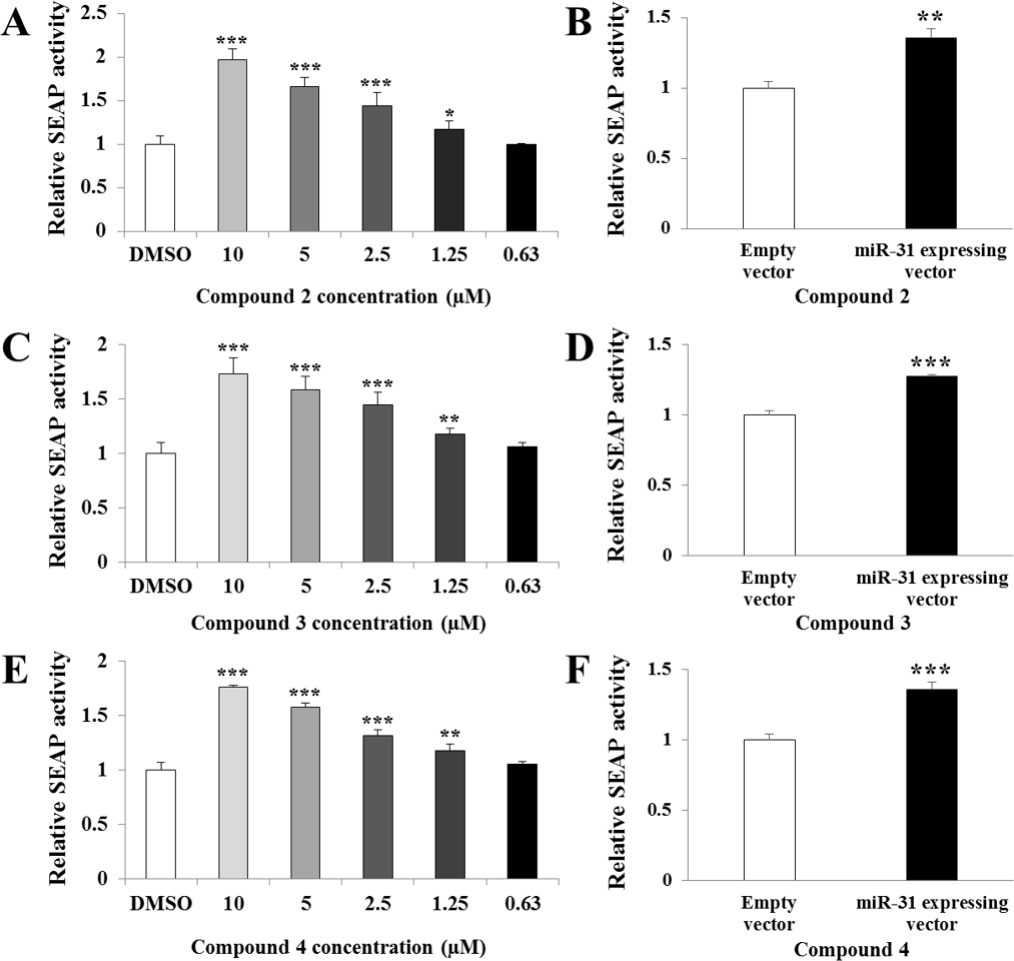
Screening of compounds with miR-31-modulating activity using the SEAP reporter assay. HEK-293T cells were co-transfected with the miR-31 expression construct (A, C, E) or empty miRNA expression construct (B, D, F) and the SEAP reporter construct containing miR-31 binding sites in the 3′UTR of the *SEAP* reporter gene and subsequently treated with the compounds for 24 h. Culture supernatants were then collected for detection of SEAP activity. In each experiment, transfection efficiency was assessed by measuring GFP produced from the miR-31 expression vector. Cells treated with compounds 2 (A), 3 (C), and 4 (E) showed a dose-dependent increase in SEAP activity in the presence of miR-31 compared to that of the DMSO-treated controls. However, compounds 2 (B), 3 (D), and 4 (F) did not increase SEAP activity without miR-31 expression. SEAP activity is presented as mean ± SD. *, p < 0.05; **, p < 0.01; ***, p < 0.001 (Student’s *t*-test).

Initial screening identified about 30 hit compounds that exhibit miR-31 inhibitory activity. To confirm the inhibitory effect of these chemicals on miR-31 expression, we selected three active compounds with common aminosulfonylarylisoxazole scaffold (compounds 2, 3, and 4) that showed the most potent miR-31-modulating activities (Fig 1) and evaluated their activities at concentrations ranging from 0.63 μM to 10 μM. As shown in Fig 2A, 2C and 2E, compounds 2, 3, and 4 inhibited miR-31 expression in a dose-dependent manner, which in turn resulted in a dose-dependent increase in SEAP activity. The observed increase in SEAP activity was not a direct effect of these compounds because SEAP activity was not increased in the absence of miR-31 expression (Fig 2B, 2D and 2F). Aminosulfonylarylisoxazole compounds are purely synthetic, and their biological functions other than miR-31 modulation have not been reported yet.

The selected compounds were then re-synthesized by coupling sulfonyl chloride **1** with various amines in the presence of tetrabutylammonium hydrogen sulfate, and their corresponding activities were re-evaluated. Cytotoxicity tests indicated >90% cell viability following treatment with the compounds at 5 μM (S1 Fig).

### Specific inhibition of miR-31 expression

**Fig 3:**
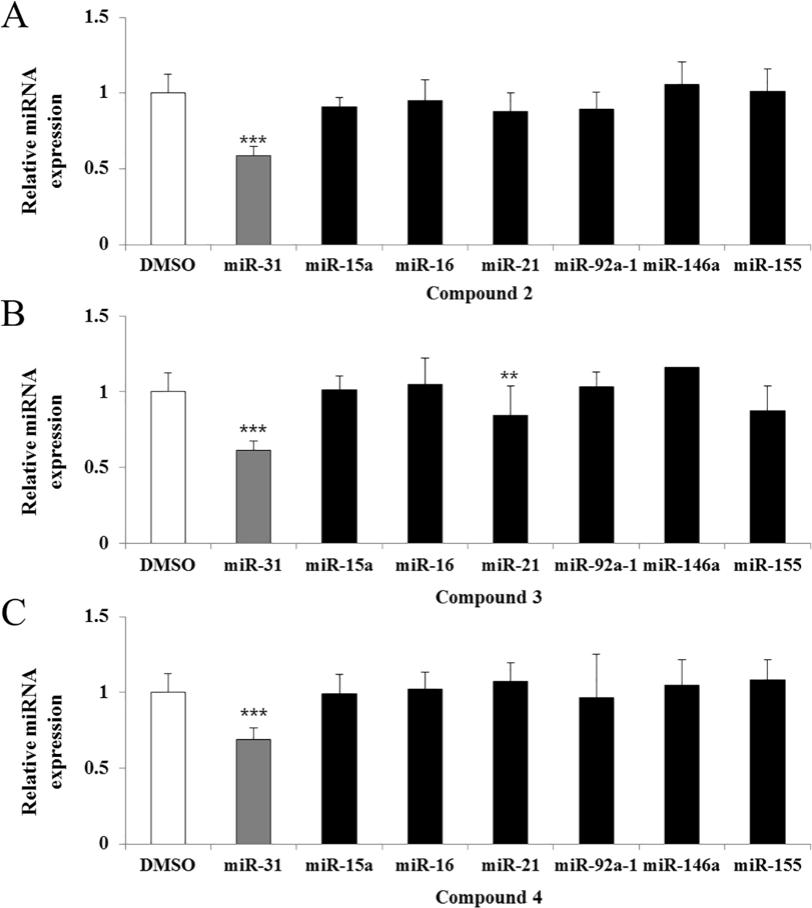
Treatment with compounds induced specific changes in miR-31 expression. Expression levels of miR-15a, 16, 21, 31, 92a-1, 146a, and 155 after treatment of A549 cells with 5 μM compounds 2 (A), 3 (B), and 4 (C) were determined via qRT-PCR. miRNA expression levels were normalized against *RNU6B*, and the values shown are relative to those of cells treated with DMSO alone. Data are representative of three independent experiments with similar results and are expressed as mean ± SD. **, *p* < 0.01; ***, *p* < 0.001.

To investigate the inhibitory effects of compounds **2**, **3**, and **4** on endogenous levels of miR-31, we measured the expression levels of miR-31 in A549 cells by qRT-PCR. As shown in Fig 3, the expression of endogenous miR-31 was significantly reduced upon treatment with compounds **2**, **3,** and **4**. In addition, we measured the expression of other miRNAs, such as miR-15a, miR-16, miR-21, miR-92a-1, miR-146a, and miR-155, to assess the selectivity of the selected compounds towards miR-31. miR-15, miR-21, miR-92a, miR-146a, and miR-155 are well-known oncogenic or tumor suppressive miRNAs that also play critical roles in tumorigenesis and tumor progression. In addition, miR-16 is frequently used to normalize miRNA levels because it exhibits high and relatively stable expression across various samples [30]. Results showed that compounds **2** and **4** specifically inhibited miR-31 without affecting the expression of the other miRNAs. On the other hand, compound **3** was found to induce slight alterations in the expression levels of both miR-31 and miR-21 (Fig 3).

### Effects of candidate miR-31 regulators on the expression of target mRNAs

**Fig 4:**
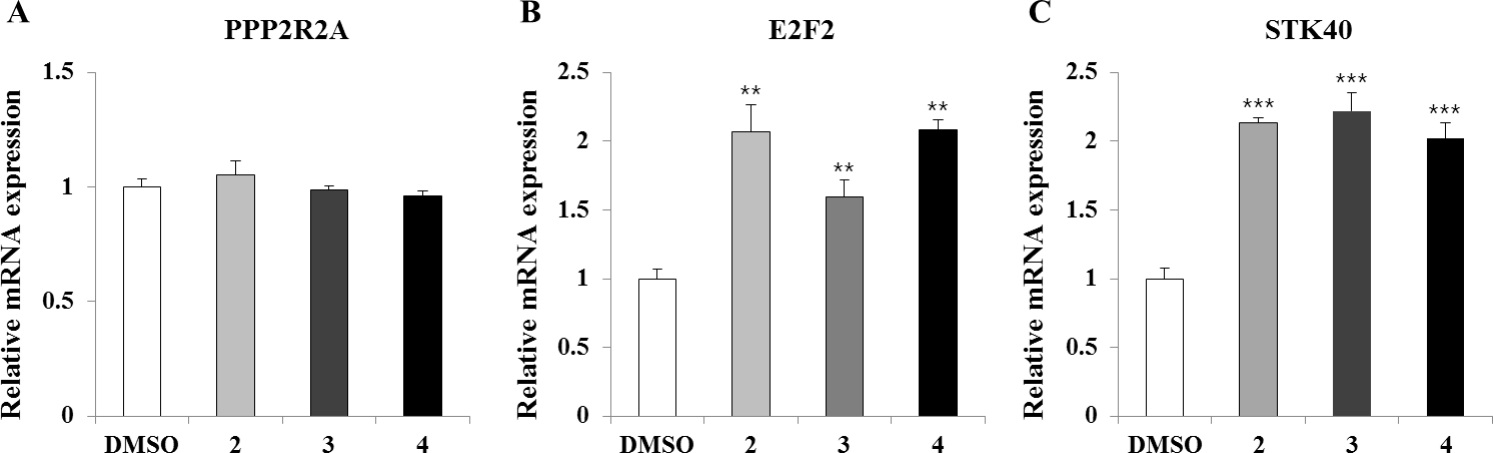
Expression of miR-31 target mRNAs following compound treatment. A549 cells were treated with compounds 1, 2, and 3 for 24 h. Total RNAs were extracted for subsequent qRT-PCR analyses of miR-31 target mRNAs. The Ct values for *PPP2R2A* (A), *E2F2* (B), and *STK40* (C) were normalized by the Ct value for *GAPDH*. Values represent mean ± SD. **, p < 0.01; ***, p < 0.001 (Student’s *t*-test) compared to expression in DMSO-treated cells.

Ectopic expression of miR-31 has been demonstrated to reduce the expression of target mRNAs, such as *STK40*, *E2F2*, and *PPP2R2A*, in A549 cells [31]. E2F2 is a tumor suppressor that inhibits the cell cycle [32], while STK40 acts as a negative regulator of NF-kB-mediated transcription [33]. PPP2R2A, also known as protein phosphatase 2A B55 subunit, has been demonstrated as a tumor suppressor that induces apoptosis [10]. In colon cancer, esophageal neoplasia, and lung cancer, miR-31 acts as an oncogenic miRNA by inhibiting the expression of *E2F2*, *STK40*, and *PPP2R2A*, respectively. Given the reciprocal expression of miR-31 and its target mRNAs, we performed qRT-PCR to explore the changes in expression of target mRNAs in A549 cells following treatment with compounds **2**, **3**, and **4**. As shown in Fig 4, treatment with the compounds upregulated the mRNA expression of *E2F2* and *STK40* but did not affect *PPP2R2A* expression.

### Upregulation of target proteins by candidate miR-31 regulators

**Fig 5:**
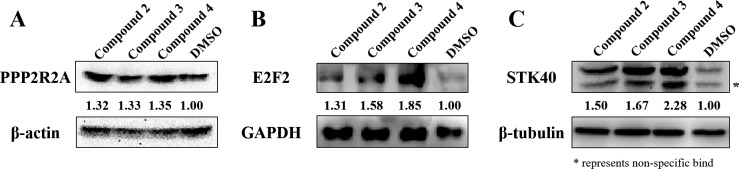
Protein expression of miR-31 targets following compound treatment. A549 cells were treated with compounds 2, 3, and 4 and lysed with RIPA buffer. Total protein concentration was calculated using a BCA assay kit, and 20 μg of total protein was used for western blot analyses of PPP2R2A (A), E2F2 (B), and STK40 (C). The intensity of each blot was normalized against that of β-tubulin. Bands were detected using the ChemiDoc™ XRS+ System. (* represents non-specific band).

miRNAs are known to regulate gene expression through multiple mechanisms. Some miRNAs mediate the translational repression of intact mRNAs, while others induce mRNA degradation. As shown in Fig 5A, cells treated with compounds 2, 3, and 4 showed higher protein expression of PPP2R2A than those of DMSO-treated controls, indicating that miR-31 represses PPP2R2A expression via a post-transcriptional mechanism. In addition, consistent with the observed increase in mRNA expression, protein expression of E2F2 and STK40 was also induced by treatment with compounds 2, 3, and 4.

### Candidate miR-31 regulators inhibited miR-31 expression by blocking the maturation from precursor miR-31 to mature miR-31

**Fig 6:**
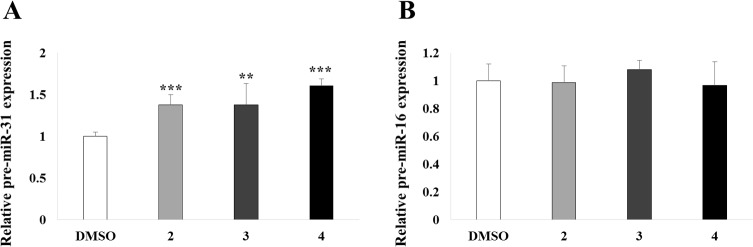
Expression of precursor miR-31 and miR-16. A549 cells were treated with compounds 2, 3, and 4, and total RNAs were extracted to detect the expression of pre-miR-31 (A) and pre-miR-16 (B). Ct values for all samples were normalized against *RNU6B*. Values represent mean ± SD. **, p < 0.01; ***, p < 0.001 (Student’s *t*-test) compared with expression in DMSO-treated cells.

To determine the mode of action of the three selected compounds, we examined the expression of precursor-miR-31 (pre-miR-31). Interestingly, we observed that pre-miR-31 expression was not decreased in treated cells but was slightly increased compared to those in the DMSO-treated controls (Fig 6A). In contrast, expression of the non-target miRNA pre-miR-16 was not affected by compound treatment (Fig 6B). These findings suggest that aminosulfonylarylisoxazole compounds block the maturation of pre-miR-31 into mature miR-31, resulting in the accumulation of pre-miR-31.

### MiR-31 mimics blocked the effects of aminosulfonyl-arylisoxazole derivatives

**Fig 7:**
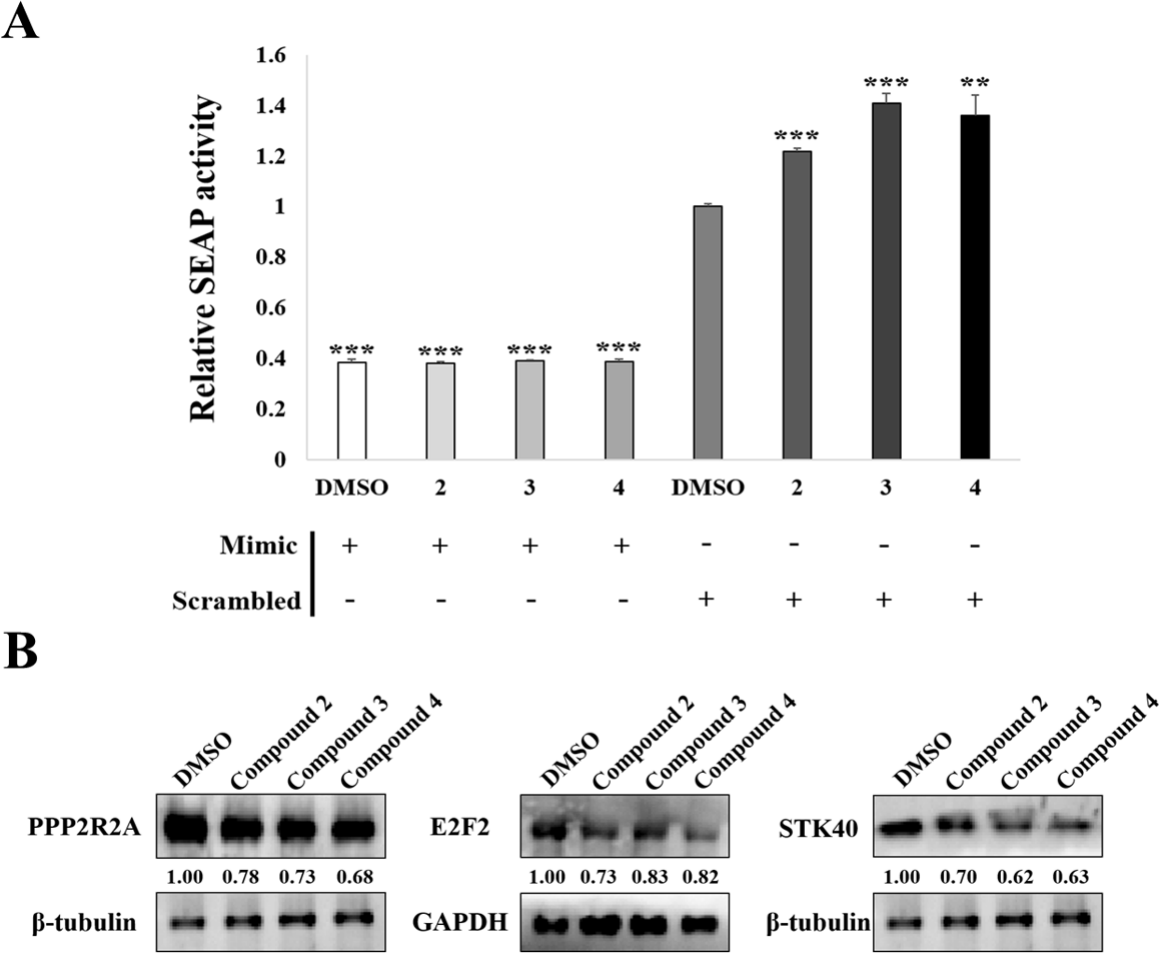
Abolition of aminosulfonylarylisoxazole derivative function by miR-31 mimics. HEK-293T cells were transfected with mature miR-31 mimics and SEAP reporter constructs containing miR-31 binding region in the 3'UTR of the reporter gene in the presence of compounds 2, 3, or 4, after which SEAP activity was measured (A). A549 cells were treated with miR-31 mimics and compounds 2, 3, or 4, and protein expression levels of PPP2R2A, E2F2, and STK40 was measured (B). Scrambled miRNA mimics were used as negative control. Data represent values from three independent experiments with similar results. Statistical significance was shown compared to DMSO control treated with scrambled miRNA mimics.

To validate that aminosulfonylarylisoxazole derivatives decreased miR-31 expression by blocking the maturation from pre-miR-31 to miR-31, SEAP assay was performed by co-transfecting HEK-293T cells with mature miR-31 mimics and SEAP reporter constructs containing the miR-31 binding region in the presence of aminosulfonylarylisoxazole derivatives. Treatment with the aminosulfonylarylisoxazole derivatives did not block the function of mature miR-31 mimics, resulting in decreased in SEAP reporter activity compared to controls transfected with scrambled miRNA mimics (Fig 7A). These results confirm that aminosulfonylarylisoxazole derivatives function by inhibiting the maturation of miR-31 from pre-miR-31.

In addition, to verify that aminosulfonylarylisoxazole derivatives enhanced the expression of miR-31 targeting mRNAs and proteins, we measured protein expression of PPP2R2A, E2F2, and STK40 in A549 cells simultaneously treated with aminosulfonylarylisoxazole derivatives and miR-31 mimics. As shown in Fig 7B, aminosulfonylarylisoxazole-induced increase in PPP2R2A, E2F2, and STK40 expression was suppressed by co-treatment with miR-31 mimics, indicating that miR-31 mimics can prevent aminosulfonylarylisoxazole compound-induced expression of target proteins.

## Discussion

In this study, efforts to identify chemical regulators of miRNAs led to the discovery of small-molecule inhibitors of miR-31, a tumor-associated miRNA. Previous studies have shown that miR-31 exerts oncogenic or tumor-suppressive effects depending on the cancer type and acts by regulating the expression of target genes involved in proliferation, differentiation, apoptosis, and metastasis [12, 13, 34–38]. In addition, miR-31 is involved in the progression of autoimmune and allergic diseases by modulating T-cell function [36, 39–42]. Thus, the restoration of miR-31 expression to normal levels serves as a potential therapeutic strategy for the treatment of miR-31-related diseases.

Aberrant expression of miRNAs can be corrected using oligonucleotide-based miRNA mimics, antisense nucleotides, and small molecule-based activators or inhibitors [20, 22–27, 43, 44]. Sequence-specific regulation of miRNAs can be achieved using oligonucleotides. The manipulation of miRNA expression by oligonucleotide-based therapeutics has been tested in several preclinical studies [45–49]. However, oligonucleotide-based drugs have certain limitations related to stability and nuclease degradation, delivery to intracellular targets, and pharmacokinetics, which impede their development as drugs. Therefore, attempts have been made to identify small molecule-based miRNA regulators that are more suitable for drug development and can be readily manufactured with the desired pharmacological and pharmacokinetic properties [50].

Using the SEAP method, we identified compounds 2 and 4, which share a common aminosulfonylarylisoxazole structure. Compounds 2 and 4 selectively downregulated miR-31 expression without affecting the expression of six other miRNAs, namely, miR-15a, miR-16, miR-21, miR-92a-1, miR-146a, and miR-155. In addition, treatment with these compounds increased both the mRNA and protein levels of the miR-31 targets, namely, PPP2R2A, E2F2, and STK40. Furthermore, treatment with these compounds reduced levels of mature miR-31, but increased the amount of its precursor form. These results imply that compounds 2 and 4 target the processing of pre-miR-31 into mature miR-31 and thus inhibit the production of mature miR-31.

Therefore, this series of aminosulfonylarylisoxazole derivatives deserve further investigation. Structural optimization of these compounds could lead to the development of more potent and highly selective miR-31 regulators.

## Supporting information

S1 Fig The effect of compounds 2, 3, and 4 on cell viability. Cell viability was expressed as the percent ratio of treatment with compounds 2–4, normalized to a 0.5% DMSO control (0 μM). Then cell viability was measured using an MTT assay. Data are representative of three experiments and were performed in triplicate. Values represent the means ± SD.
(TIF)

S2 Fig Relative endogenous expression of different miRNAs in A549 cells. Endogenous expression levels of miR-15a, 16, 21, 31, 92a, 146a, and 155 in A549 cells were determined by qRT-PCR. miRNA expression levels were normalized against *RNU6B*. Values shown are relative to miR-31 expression levels.
(TIF)
