# Supplementary figures and images for: Identification of aminosulfonylarylisoxazole as microRNA-31 regulators
Source: PLoS One. 2017 Aug 4;12(8):e0182331. doi: 10.1371/journal.pone.0182331 (PMC5544221; doi:10.1371/journal.pone.0182331)

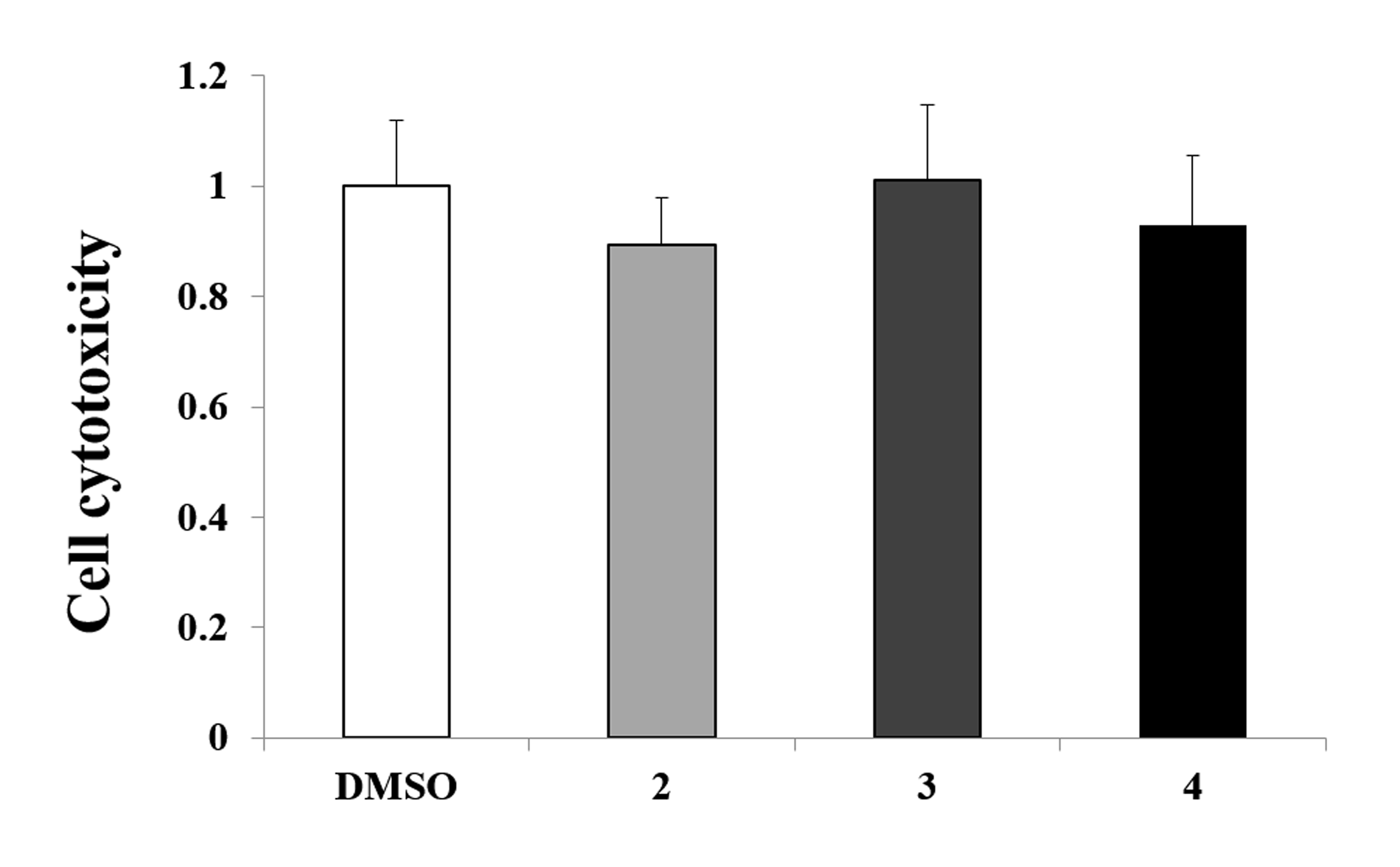

Supplement: S1 Fig — Cell viability was expressed as the percent ratio of treatment with compounds 2–4, normalized to a 0.5% DMSO control (0 μM). Then cell viability was measured using an MTT assay. Data are representative of three experiments and were performed in triplicate. Values represent the means ± SD. (TIF) [file pone.0182331.s001.tif]

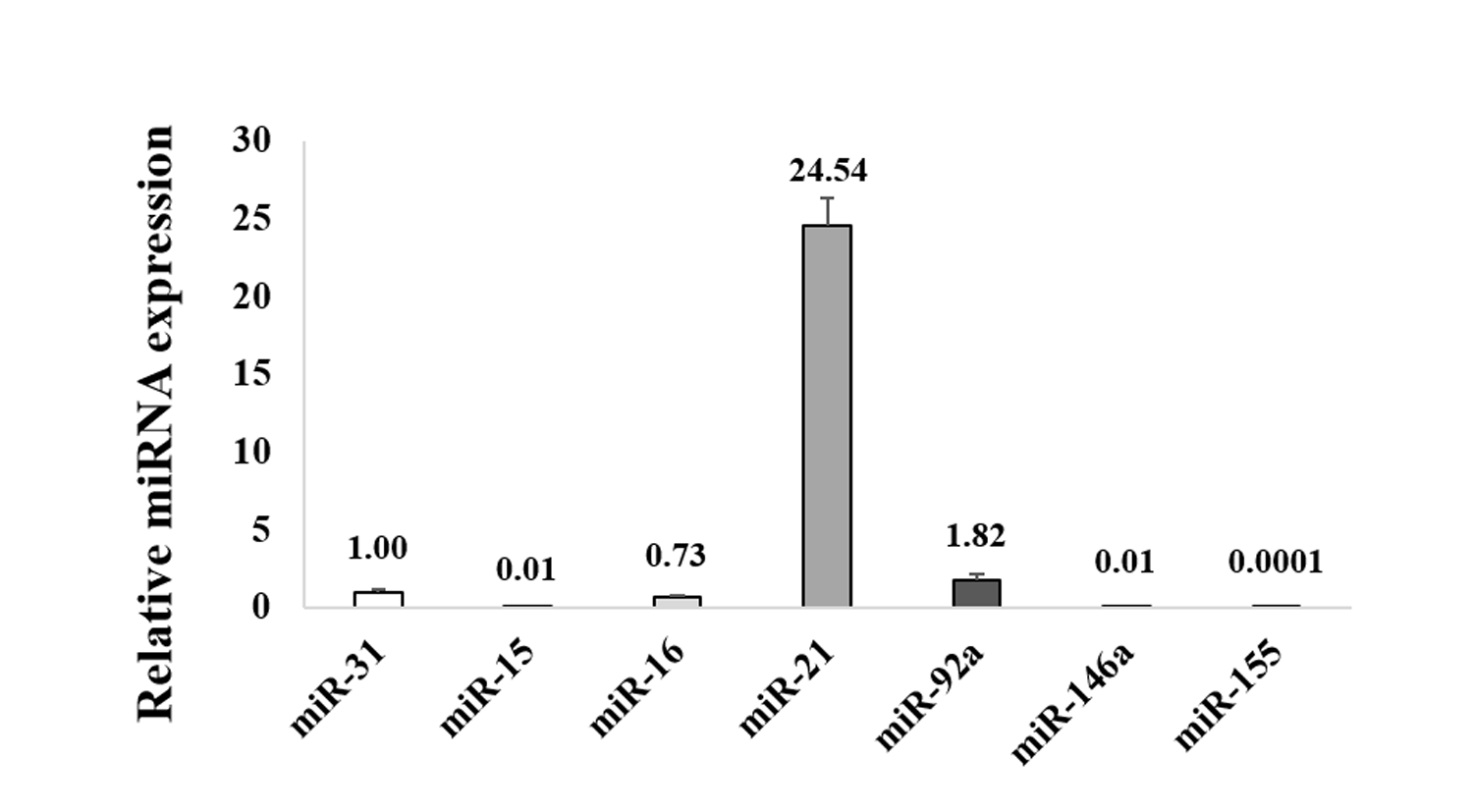

Supplement: S2 Fig — Endogenous expression levels of miR-15a, 16, 21, 31, 92a, 146a, and 155 in A549 cells were determined by qRT-PCR. miRNA expression levels were normalized against RNU6B. Values shown are relative to miR-31 expression levels. (TIF) [file pone.0182331.s002.tif]
